# H1N1 Infection-Related Hemophagocytic Lymphohistiocytosis in a Child

**DOI:** 10.4274/Tjh.2013.0022

**Published:** 2013-12-05

**Authors:** Fatih Demircioğlu,, Elif Kazancı, Dildar Bahar Genç, Hakan Erdoğan, Sevil Bilir Göksügür, Mervan Bekdaş

**Affiliations:** 1 Medical Faculty, Abant İzzet Baysal University, Department of Pediatrics, Bolu, Turkey; 2 Dörtçelik Children’s Hospital, Department of Pediatrics, Bursa, Turkey

**Keywords:** children, Hemophagocytic lymphohistiocytosis, Swine-origin influenza

## TO THE EDITOR

Hemophagocytic lymphohistiocytosis (HLH) is a clinical condition characterized by macrophage and activated histiocyte proliferation, leading to uncontrolled phagocytosis of hematopoietic precursor cells. The clinical presentation is characterized by fever, pancytopenia, hepatosplenomegaly, and hemophagocytosis in the reticuloendothelial system. In addition to the primary form of the disorder, secondary HLH has been associated with a variety of infections, malignancy, and autoimmune disease [1]. Virus-associated HLH is a well-recognized clinical condition. Most cases are related to Epstein-Barr virus (EBV), cytomegalovirus (CMV), human herpes virus 6, and human herpes virus 8 infections [2]. H1N1 influenza-associated HLH has been reported in children extremely rarely [3,4]. We present here a successfully treated case of severe H1N1 influenza-related HLH. 

A 4-year-old boy was admitted to our hospital with a 5-day history of fever, malaise, myalgia, cough, respiratory distress, oliguria, and diffuse petechial eruption. On physical examination he was found febrile, pale, and dehydrated. His vital signs included a temperature of 38.7 °C, heart rate of 154 beats per minute, respiratory rate of 42 breaths per minute, blood pressure of 79/56 mmHg, and capillary refill time of >2 s. He had 6-cm hepatomegaly and 9-cm splenomegaly below the costal margin. The rest of the physical examination was normal. 

Laboratory examination demonstrated hemoglobin of 9.2 g/dL, white blood cell count of 3000/mm3 (24% neutrophils, 62% lymphocytes, and 14% monocytes), and platelet count of 56,000/mm3. In the peripheral smear investigation, toxic granulation was observed. Biochemical evaluation revealed that blood urea nitrogen was 118 mg/dL, creatinine 1.6 mg/dL, sodium 124 mmol/L, potassium 2.92 mmol/L, C-reactive protein 18.5 mg/dL (N: 0.0-1.0 mg/dL), fibrinogen 120 mg/dL, and ferritin 875 ng/mL. Serum triglycerides were increased to 268 mg/dL but cholesterol levels were in the normal range. The serum aspartate aminotransferase, alanine aminotransferase, and lactate dehydrogenase levels were elevated at 208, 72, and 893 U/L, respectively. Serologic markers for EBV; CMV; parvovirus B19; toxoplasmosis; hepatitis A, B, and C viruses; human immunodeficiency virus, salmonella; and Brucella agglutinins were all negative. Results of the urine analysis were in the normal range. Chest radiograph demonstrated infiltrates in both lung fields and bilateral pleural effusions.

**Figure 1:**
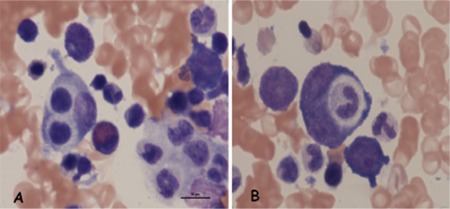
Bone marrow smear shows histiocytes with hemophagocytosis (A and B).

Intravenous fluid replacement and meropenem and dopamine infusion were started. On the second day of hospital admission, H1N1 influenza was identified from nasopharyngeal swabs by specific PCR, and then oseltamivir (6 mg/kg in 2 daily doses, per os) was added to the treatment. HLH was suspected because of the patient’s persistent fever, severe pancytopenia, hyperferritinemia, hypertriglyceridemia, hepatosplenomegaly, and hypofibrinogenemia. A bone marrow aspiration was performed and the results revealed typical hemophagocytosis (Figure 1). Therefore, intravenous immunoglobulin (IVIG) was added to the treatment due to the diagnosis of H1N1-associated HLH. Renal functions returned to normal on the second day of treatment. The patient’s respiratory function gradually recovered on day 4. On the third day of oseltamivir and meropenem therapy, the patient became afebrile, and oseltamivir and meropenem therapies were stopped after 5 days and 10 days of treatment, respectively. On the 10th day of admission, physical examination and laboratory evaluation were found normal. He was discharged on day 14. In the first year of follow-up, hematologic and biochemical values were normal, except for moderate splenomegaly and hyperferritinemia (ferritin level was 725 ng/mL). Immunodeficiency, infection, malignancy, and other splenomegaly-related conditions were excluded. Informed consent was obtained. 

## DISCUSSION

The H1N1 virus was first observed in March 2009 in Mexico and then spread rapidly throughout the world. Cases of H1N1 have generally been mild, with patients recovering fully within 1 week [5]. Hematological manifestation of H1N1 has commonly been observed with leucopenia, neutropenia, and idiopathic thrombocytopenic purpura. Rarely, HLH has been observed [6].

HLH represents a severe hyperinflammatory condition with the major symptoms of prolonged fever, peripheral cytopenia affecting at least 2 lineages, hepatosplenomegaly, hypertriglyceridemia and/or hypofibrinogenemia, hyperferritinemia, decreased or absent natural killer cell activity, and high soluble interleukin 2 receptor serum levels and hemophagocytosis by activated macrophages. Five of these 8 criteria should be fulfilled to confirm diagnosis [1,2]. Our patient had prolonged fever, pancytopenia, hepatosplenomegaly, hyperferritinemia, hypofibrinogenemia, and hemophagocytosis at bone marrow aspiration and was diagnosed with HLH according to the criteria of HLH 2004 [1,2].

Our patient had prolonged fever, fatigue, myalgia, pneumonia, and prerenal insufficiency and diagnosed H1N1 influenza. The first reported case of H1N1 influenza virus-related HLH was in a 17-year-old female patient who completely recovered with steroid and oseltamivir treatment [3]. Following this case report, 23 more patients were reported of various ages between 2 months and 61 years old. In most of the cases, pancytopenia, hepatosplenomegaly, hyperferritinemia, hypofibrinogenemia, and hemophagocytosis at bone marrow aspiration were detected [3,4,7,8,9,10,11,12]. Oseltamivir and systemic steroid was the most commonly preferred treatment option. We diagnosed our patient with H1N1 virus-associated HLH. Besides oseltamivir, we also add IVIG to the treatment. On the third day of oseltamivir and second day of IVIG therapy, the patient became afebrile; oseltamivir therapy was stopped after 5 days of treatment.

HLH has high mortality rates if early diagnosis and treatment are delayed. In previously reported cases, only 5 out of 23 patients survived. In our case, treatment was started at the second day of hospitalization and a good response was achieved. In the first year of follow-up, hematologic and biochemical values remained normal, except for moderate splenomegaly and hyperferritinemia. To et al. reported prolonged viral load clearance and cytokine activation in H1N1 swine influenza virus infection [13]. We did not detect any obvious cause of splenomegaly in our patient. The splenomegaly and hyperferritinemia in our case may therefore be associated with prolonged inflammatory process and decreased viral load clearance. We report a very rare case of H1N1-related HLH in childhood, which was diagnosed early and successfully treated. If it is diagnosed late, H1N1-associated HLH has very high mortality rates, but early diagnosis and treatment have favorable outcomes. It may also cause prolonged viral load clearance and cytokine response. 

## CONFLICT OF INTEREST STATEMENT

The authors of this paper have no conflicts of interest, including specific financial interests, relationships, and/ or affiliations relevant to the subject matter or materials included.
